# Lessons From the Portuguese Solid Organ Donation and Transplantation System: Achieving Success Despite Challenging Conditions

**DOI:** 10.3389/ti.2023.11008

**Published:** 2023-05-25

**Authors:** Simon Streit, Charlotte Johnston-Webber, Jasmine Mah, Apostolos Prionas, George Wharton, Jorge Paulino, Ana Franca, Elias Mossialos, Vassilios Papalois

**Affiliations:** ^1^ Department of Health Policy, London School of Economics and Political Science, London, United Kingdom; ^2^ Department of Medicine, Dalhousie University, Halifax, NS, Canada; ^3^ Department of Surgery, Imperial College, London, United Kingdom; ^4^ Department of General Surgery, Whipps Cross Hospital, Barts Health NHS Trust, London, United Kingdom; ^5^ Hepato-Biliary-Pancreatic and Transplantation Centre, Curry Cabral Hospital, Lisbon, Portugal; ^6^ Instituto Português do Sangue e da Transplantação, Lisbon, Portugal; ^7^ Institute of Global Health Innovation, Imperial College, London, United Kingdom; ^8^ Renal and Transplant Unit, Hammersmith Hospital, Imperial College Healthcare NHS Trust, London, United Kingdom

**Keywords:** organ donation, organ transplantation, transplantation policy, Portugal, transplant program

## Abstract

Over the past two decades, Portugal has become one of the world leaders in organ donation and transplantation despite significant financial constraints. This study highlights how Portugal achieved success in organ donation and transplantation and discusses how this information might be used by other countries that are seeking to reform their national programs. To accomplish this goal, we performed a narrative review of relevant academic and grey literature and revised our results after consultation with two national experts. Our findings were then synthesized according to a conceptual framework for organ donation and transplantation programs. Our results revealed several key strategies used by the Portuguese organ donation and transplantation program, including collaboration with Spain and other European nations, a focus on tertiary prevention, and sustained financial commitment. This report also explores how cooperative efforts were facilitated by geographical, governmental, and cultural proximity to Spain, a world leader in organ donation and transplantation. In conclusion, our review of the Portuguese experience provides insight into the development of organ donation and transplantation systems. However, other countries seeking to reform their national transplant systems will need to adapt these policies and practices to align with their unique cultures and contexts.

## Graphical Abstract

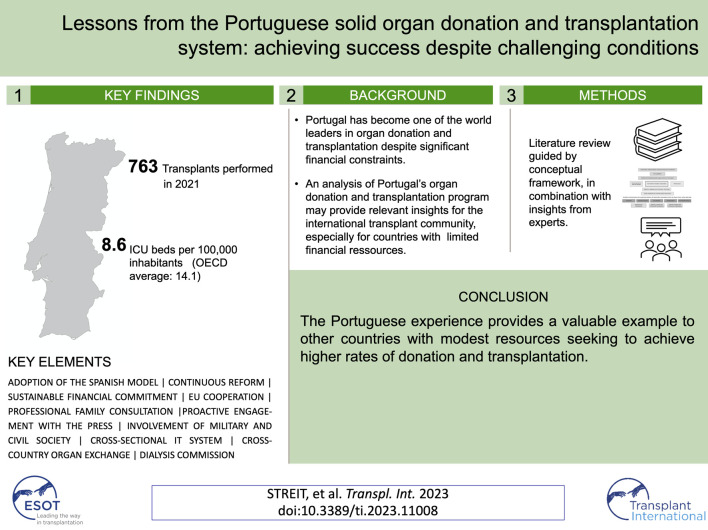

## Introduction

Efforts to develop a high-functioning organ donation and transplantation program require specific investments, including those focused on reimbursement of staff and facilities, infrastructure, robust governing structures, research and development, and digital information technology (IT) (1). However, organ donation and transplantation rates are not always directly related to the financial resources available to a given healthcare system (2). This point is clearly illustrated by the example of Portugal, which as a country spends slightly less of its gross domestic product (GDP) on healthcare and significantly less per inhabitant than the European average (3). Although Portugal, prior to the COVID-19 pandemic, supported fewer critical care beds (an important element of the infrastructure required for a successful organ donation and transplantation program) than any of the other European nations (i.e., only 4.2 beds per 100,000 inhabitants (4), it has substantially increased the rates of organ donation during the last two decades to cope with a significant burden of patients with organ failure including a high number of patients with chronic kidney disease and patients on renal replacement therapy ((5); Table 1). Before the disruptions resulting from the COVID-19 pandemic, Portugal reached 33.7 deceased donations per million population (pmp) (6). Thus, Portugal not only outperforms other Southern European countries with similar population sizes, demographics, and economic constraints (e.g. Greece), it has become a world leader in deceased donation, ranking second in Europe and third worldwide in 2018 (6). Therefore, previous reviews of the Portuguese system have been used as a basis for national policy reform and an in-depth study of Portugal’s organ donation and transplantation program may provide highly relevant insights for the international transplant community (7).

**TABLE 1:** Health system financing and population health in Portugal: key statistics.

Health system	References
• A universal taxpayer-funded health system that co-exists with special health insurance schemes that apply to particular professionals and private voluntary health insurance	(3)
• Amount spent on healthcare *per capita*, 2,314€; 9.5% of the gross domestic product (GDP)	(3)
• Public spending as a percentage of the total health expenditure: 61%	(3)
• Out-of-pocket payments as a percentage of the total health expenditure: 30.5%	(3)
• Percentage of the population reporting an unmet need for medical care: 2.7%	(3,8)
**Health status**	
• Percentage >65 years of age: 22.1%; EU average, 20.6%	(3)
• Life expectancy: 81.1 years; EU average, 80.6 years	(3)
• Smoking (% of the population who are daily smokers): 14.2%; OECD average, 16.5%	(9)
• Alcohol (liters consumed *per capita* per year): 10.4 L; EU average, 20.6%	(9)
• Percentage overweight or obese (BMI >25): 67.6%; OECD average, 56.4%	(9)
• Patients maintained on renal replacement therapy: prevalence, 2008.4 per million population (pmp); incidence, 250.7 pmp	(10)
• Age-standardized prevalence of chronic kidney disease: 5.4%; (global 8.7%)	(11)

EUR, Euro; EU, European Union; OECD, Organisation for Economic Co-operation and Development; BMI, body mass index.

The paper aims to provide an updated review of the critical features of the Portuguese program that have led to its status as a world leader in organ donation and transplantation The goal is to describe and discuss insights that might be of use to other countries, notably those that share similar demographics and financial constraints.

## Materials and Methods

Our report on the Portuguese transplant program is based on an in-depth study that was initially designed to provide a comprehensive review of the organ donation and transplantation program in Greece (12). As part of the original report, we identified key features and critical factors that contributed to the success of the Portuguese transplant system, a country with similar demographic and financial resources to Greece. Special emphasis was placed on the question of how this level of success was achieved despite substantial demographic and financial challenges. Here, we present, update, and discuss the major findings from this report.

We began by conducting a narrative literature review of relevant academic and grey literature, including published reviews that discussed the Portuguese organ donor and transplant system. We performed a search of relevant websites including those maintained by the Portuguese government, the Ministry of Health, and the Instituto Portugues do Sangue e da Transplantação; policy reports that focused on donation reform in Portugal were retrieved *via* a Google search. An additional database search was then performed using Medline, Web of Science, and Google Scholar to identify relevant academic literature.

We then reviewed our findings with two experts on the Portuguese organ donation and transplantation program (coauthors Dr Jorge Paulino and Dr Ana Franca). These experts were each invited to present key features of the Portuguese transplant system in an online interview. They also answered questions and provided feedback and additional materials such as national statistics or legislative documents.

**FIGURE 1:**
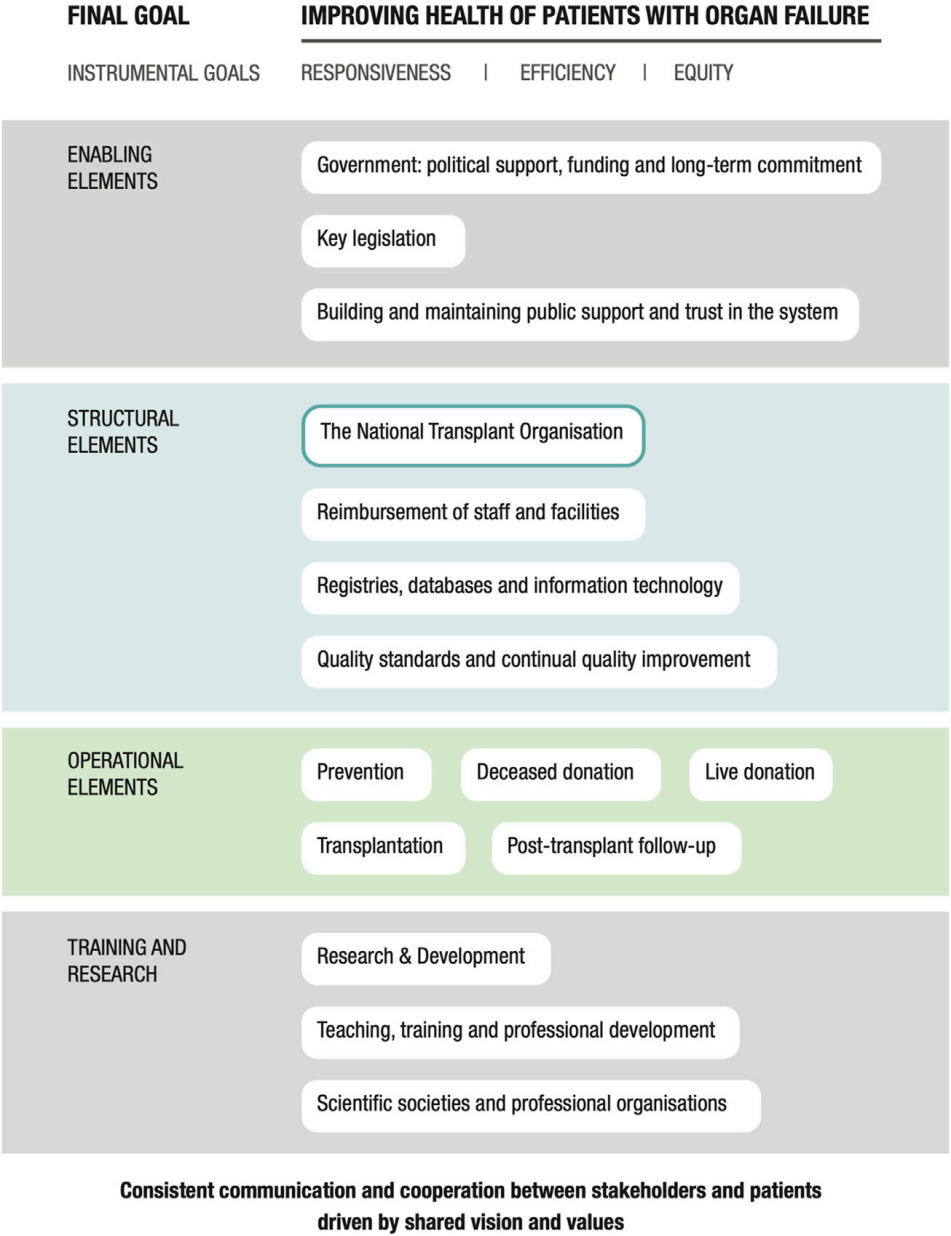
A conceptual framework for understanding a national solid organ donation and transplantation program: the essential building blocks (1).

In the following sections, we first outline the main advances and achievements of the Portuguese transplant system. We then describe key elements and insights provided by the Portuguese organ donation and transplantation program. These insights are presented according to the domains of a conceptual framework for transplant systems described by Johnston-Webber et al. as shown in Figure 1 (1). To synthesize key lessons from a well-functioning system, we mainly focus on the time period from initial development of the transplant system in 1993 up to 2019. In addition, we provide an overview of recent developments including adaptations that were made in response to the Coronavirus disease 2019 (COVID-19) pandemic with the latest data available from 2021. Finally, we discuss factors that may have played a role in Portugal’s successful adaption of the Spanish organ donation and transplantation model.

According to Johnston-Webber et al. (1), the main goal of an organ donation and transplantation system is to improve the health of patients with organ failure by successful prevention of organ failure, facilitating organ donation, performing safe and effective transplantation, and providing suitable follow-up. Transplant systems must be responsive to the needs of patients, donors, and the general population, and must be capable of using their resources efficiently and providing equitable access to care. The multiple components of this system will need to interact effectively with one another to achieve these goals.

## Results

### Context and Trends Identified in the Portuguese Transplant System

**FIGURE 2:**
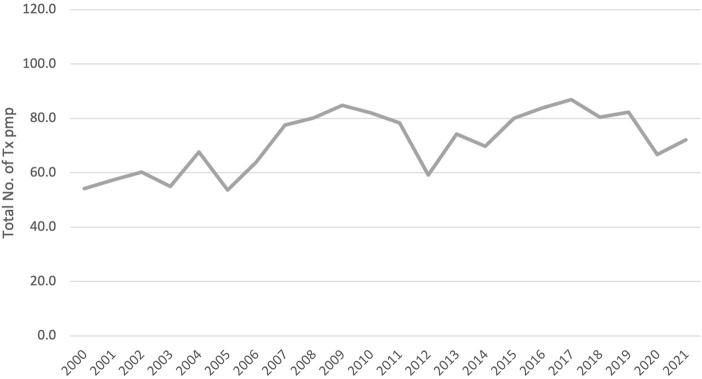
Development of Transplantation Activity in Portugal. *Data of the WHO-ONT Global Observatory on Donation and Transplantation (5). Tx, Transplants; pmp, per million population.

As has been described for other successful programs, the current Portuguese system is the result of many years of planning, development, and review. An overview of the main advances over the past few decades will be helpful to build an understanding of the structure of the program as it stands today. Table 2 summarizes the most important steps Portugal has undertaken since 1993 to build a sustainable and effective organ donation and transplantation program. Figure 2 illustrates the development of transplantation activity in Portugal for the past 20 years.

**TABLE 2:** Main developments in Portugal’s organ donation and transplantation program over the past 30 years (7, 13, personal correspondence).

1993	Establishment of a national legal framework for organ donation and transplantation
1993	Establishment of the first national institution (Organização Portuguesa de Transplantação) and five regional authorities dedicated to organ donation and transplantation
1994	Implementation of a national non-donor registry
1996	Implementation of regional transplant coordinators
2007	Introduction of hospital donor coordinators
2008	Implementation of dedicated training programs designed for healthcare professionals in the field of organ donation and transplantation
2008	Restructuring of care for patients with end-stage renal disease
2009–11	Engagement in a set of European Union-wide collaborations to promote organ donation and transplantation
2012	Restructuring and creation of the current national transplant organization (Instituto Portugues do Sangue e da Transplantação [IPST])
2013	Implementing a legal framework for donation after circulatory death (DCD)
2015	Establishment of protocols for the protection of living organ donors (Decree-Law No. 168/2015)
2019	Establishment of July 20th as the National Day of Organ Donation and Transplantation
2019	Introduction of informed consent policy for post-mortem organ and tissue donation by foreign citizens who have not established permanent residence in Portugal
2020	Regulation of the structure and responsibilities of the National Dialysis Commission

### Key Elements and Policies Leading to Transplant Reform

The following sections provide an overview of the key features of the Portuguese program identified in this study as playing important roles in promoting its success (Table 3).

**TABLE 3:** Key features from the Portuguese transplant system that might be adopted by other countries.

Framework domain	Key features	Details
Enabling Elements: Government: Political Support, Funding, Long-Term Commitment, and Key Legislation	A set of basic reforms can have an immediate and substantial impact	Portugal has modeled its program on that of neighboring Spain. Key elements of this program include the establishment of a robust National Transplant Organization (NTO) with regional offices and the employment of specifically-trained organ donor coordinators in hospitals who have ongoing access to high-quality educational programs
Enabling Elements: Building and Maintaining Public Support and Trust in the System	Active inclusion of state institutions and civil society in the process of organ donation reform	Portugal has included a wide range of stakeholders and civil society in ongoing consultations regarding the development of the program. This has helped to raise the profile of organ donation and transplantation, gain the support of the public, and resolve complex ethical issues and religious concerns
Structural Elements: National Transplant Organization	Drawing strength from collaborations	Effective use of European resources has been of great benefit to the Portuguese program. Examples of these collaborations include the creation of an international quality assurance program, training partnerships, and organ exchange schemes
Structural Elements: Reimbursement of Staff and Facilities	Continuous financial support is crucial to ensure consistent donation rates	In 2007, Portugal increased financial support for organ donation with positive results. However, this support was reduced by 50% following the economic crisis which resulted in a parallel reduction in donation rates
Structural Elements: Registries, Databases, and Information Technology (IT)	A national IT system supports complex workflows	Portugal has established an excellent digital donation and transplantation registry which operates in real-time and thus facilitates rapid communication of vital information. This registry supports all aspects of organ donation and transplant and facilitates the smooth and efficient functioning of the program
Operational Elements: Prevention	Preventative policies that address chronic kidney disease will be one way to target demand	As Portugal has very high rates of end-stage renal disease (ESRD), it has established an effective program designed to improve the management of chronic kidney disease (CKD), notably in the field of dialysis care. Prospective capitated reimbursements for dialysis have been introduced that are contingent on regular submission of quality and outcomes findings including patient survey data

### Enabling Elements

#### Government and Key Legislation

##### Continuous Reform Efforts Influenced by the Spanish Transplant Model Are a Promising Way to Build a Well-Functioning Transplant System Over Time

The Portuguese program was initiated in 1993 and is broadly based on the Spanish model which has been the source of many of its key features (7).

By law, “all national citizens, stateless persons, and foreigners residing Portugal who have not declared their status as non-donors to the Ministry of Health are potential post-mortem donors.” (13–15). Citizens who do not want to be organ donors can declare their will *via* a dedicated registry for non-donors which is maintained by the Ministry of health (16). However, in practice, even where the donor’s consent is presumed or explicitly given, families are always consulted, informed and asked for agreement for donation (19, personal correspondence). Thus, consent policy in Portugal can be categorized as “soft opt-out.”

In 1993, Portugal implemented the national transplant body which included five regional offices (7). Following several cycles of restructuring (see Table 2), the Instituto Português do Sangue e da Transplantação (IPST) was able to provide regulatory oversight and coordination of the donation and transplantation processes, while the regional offices were designated as responsible for the coordination of organ retrieval, allocation, and transportation (7, 13).

The role of hospital-based organ donation coordinators was adapted from the Spanish model and introduced in 2007 (7, 15, 18, 19). Today, all participating hospitals have appointed donor coordinators from their clinical staff who are specially trained in organ donor detection and evaluation. Donation coordinators are typically medical doctors, preferably with a background in intensive care medicine and receive a monthly €500 to €1,000 additional compensation from their local hospitals for their responsibilities. They identify potential organ donors, consult with families, initiate logistics of organ donation in cooperation with the national procurement and transplant coordination network, document the donation process, promote organ donation in their local hospitals and report to hospital management and the national transplant organization (19). These staff members also have access to ongoing training and professional development opportunities *via* national and international schemes that build on existing resources such as the Donation and Transplantation Institute - Transplantation Procurement Management (DTI-TPM) program, Barcelona (7, 20).

Largely due to the changes implemented throughout the 2000s combined with clear legislation and guidance for the diagnosis of brain death (17, 21), Portugal greatly increased its rates of donation and transplantation (7, Table 2). The capacity to build on some of the core features of the Spanish system together with a focus on improving rates of donation after brain death facilitated immediate and substantial improvements in the performance of Portugal’s transplant system.

#### Building and Maintaining Public Support and Trust in the System

##### Professional Family Consultations, Proactive Engagement With the Press and Broad Involvement of Stakeholders From Government and Civil Society Have Helped to Build Trust in the System

In Portugal, the public’s willingness to donate their own, or a deceased family member’s organs has been shown to be higher than in other European countries (22-24). Additionally, relatively low numbers of people cite mistrust in the transplant system as a reason for their unwillingness to consider organ donation (24). Finally, in 2020, donations from only four of 412 potential donors (only 0.97% of the total potential donor pool) were unsuccessful because of family refusal (personal correspondence). This percentage has remained fairly stable over recent years and represents an outstanding level of success compared to results reported by many other countries worldwide (25).

Together, these findings suggests that there is a generally favorable attitude towards donation, a widespread public acceptance of the current transplant program and the ability to translate these favorable attitudes into consent for organ donation.

Several factors have played a role in achieving this success. First of all, measures that have been crucial in the Spanish context have been adopted in Portugal. This includes collaborations with the press (including training sessions for journalists) and training organ donation coordinators in conducting family consultations (7, 14, 15). The latter is especially important, as experience and training have been directly linked to the decision of families to donate organs (26). Family consultations offer the opportunity to comprehensively inform families about the clinical setting and legal situation. This prevents mistrust in a system in which, from a legal standpoint, donation could be pursued even if there is no documented will of the deceased to donate organs.

Second, The Portuguese authorities have consistently endeavored to include the public and stakeholders from civil society such as religious groups or patient organizations to dispel misconceptions regarding organ donation and win the trust of the public (7, 27). The regional procurement offices collaborate with the Portuguese Air Force (28) and the police (the National Republican Guard, or GNR) to facilitate the transportation of organs. The use of this strategy has increased the visibility and status of organ donation throughout Portugal. The GNR published a statement in conjunction with National Organ Donation Day (July 20th) that highlighted its efforts and beneficial role in this process:

“The quality and safety of organ transplantation depends on the time required for transport, a factor that is crucial in this mission. Therefore, it is the responsibility of the GNR, and in respect of security conditions, to reach the destination in the shortest time possible, thus contributing to saving yet another life. In this noble mission, this year 2021 alone, the GNR has already carried out 135 transportations of organs, involving 271 soldiers, having covered approximately 37 342 kilometres. In 2020, the GNR transported 240 organs, engaged 478 soldiers and covered 64,133 kilometres” (29).

Together, these measures have helped to gain the trust of the general public in Portugal.

### Structural Elements

#### The National Transplant Organization

##### International Collaboration can Help With the Process of Acquiring Additional Resources

Portugal has maintained several fruitful international collaborations in the field of organ donation and transplantation. Portugal has participated in multiple EU-funded programs that were included as part of the “EU Action Plan on Organ Donation and Transplantation” (18). Specifically, Portugal has improved its quality assurance system based on a European analysis of end-of-life care practices and an EU-funded indicator-driven quality improvement initiative (30-32). Moreover, the EU-wide “train the trainers” program led by TPM has helped to build local training capacity (20,33). Portugal also participates in a set of cross-organ exchange schemes with Spain and other Southern European countries, including the use of shared lists of those awaiting lung, renal, and liver transplantation (17, 34, 35). Thus, Portugal has successfully managed to compensate for its limited resources by optimizing the advantages associated with membership in the EU as well as its proximity to Spain and other Southern European countries.

#### Reimbursement of Staff and Facilities

##### Consistent Financial Commitment is Necessary to Support Structural Reform

In the 2000s, donation rates in Portugal increased steadily along with a set of structural reforms that included general investment in the donation and the transplantation sector, and the introduction of reimbursement for relevant donation activity (7). Unfortunately, this trend was reversed due to the austerity measures that were implemented in Portugal to cope with the 2008 worldwide financial crisis (7). The general impact of these measures on healthcare staff and patients, including reductions in the salaries of hospital staff and the introduction of user charges have been considered in previous publications (36, 37). Funding for donation activities was effectively cut in half (7). Donation activity decreased accordingly, thus illustrating the importance of continuous financial commitment to ensure the sustainability of structural reforms.

#### Registries, Databases, and Information Technology (IT)

##### A Cross-Sectional IT System can be Used to Manage Complex Clinical Pathways

The Portuguese transplant authorities have successfully implemented a multi-functional IT platform that supports clinical pathways associated with organ donation and transplantation (41; personal correspondence). The IT platform provides access to a consent registry, patient records (clinical information, imaging, and other data), and a real-time clinical workflow system that can be used to document, illustrate, and provide timing for all relevant clinical processes from organ procurement to transplantation. Thus, this IT platform provides critical support for healthcare professionals as it facilitates rapid and safe communication between all clinicians involved in this process. The platform also helps the transplant authorities to track and document critical processes and serves as a tool to support quality improvement and biovigilance. The data collected can also be used for research purposes (41; personal correspondence).

Taken together, the Portuguese system illustrates the advantages of implementing robust IT systems to support organ donation and transplantation. The Portuguese experience suggests that the investment needed to establish a functional digital infrastructure will be worth the costs as it will ultimately facilitate the management of complex clinical protocols.

### Operational Elements

#### Prevention

##### Concerted Efforts Toward Tertiary Prevention Pay off

Despite its success in providing kidney transplants, Portugal is currently home to the highest percentage of patients maintained on renal replacement therapy in all of Europe (39). In response to this problem, Portuguese transplant authorities have joined forces with other government agencies, medical, nursing and pharmacists associations, renal patient groups and dialysis centers to implement a dedicated dialysis commission tasked with monitoring and improving this situation (40). Toward this end, a set of reforms direct at patients with end-stage renal disease (ESRD) have been implemented (38). First, the financing of dialysis care has been shifted from a fee-for-service scheme to capitated payments in an effort to reduce costs. Second, a quality improvement initiative has been implemented that requires the collection of a set of clinical quality indicators for dialysis care as part of the prerequisite for reimbursement (38). Additionally, a patient survey focused on the quality of care has been introduced (38). Finally, copayments have been decreased and the rates of referral from general to specialist care have improved (38). Taken together, these measures resulted in an overall reduction of public payments. This was accompanied by improvements in the quality of care as indicated by the results of patient satisfaction surveys and international performance metrics for dialysis care (38, 41).

Our results suggest that countries seeking to reform their national programs might take demand-side measures into account and focus concerted efforts toward improving the care of patients who might otherwise eventually require an organ transplant. As illustrated by the Portuguese example, these efforts not only help to improve patient care but will also result in reduced costs for the healthcare system.

#### Recent Developments

##### Recent Progress on Living Donation and Donation After Circulatory Death (DCD)

In recent years, Portugal has aimed to increase the rates of living donation and donation after circulatory death (DCD) as a way to complement its successful deceased donation after brain death program.

All living donation programs will need to prioritize the implementation of measures that safeguard donor rights and psychosocial wellbeing. In Portugal, a protocol was established in 2015 that included a psychiatric evaluation both before and after live donation (42). This created a unique regimen designed to protect and support the rights of living organ donors. The protocol includes a mandatory living organ donor life insurance guarantee which covers all possible complications that might arise as a result of the donation and procurement process as well as a set of benefits provided to the donor in the event of death, the development of any level of permanent disability, or hospitalization (43). Living donation rates before the COVID-19 pandemic were at 7.28 pmp in 2019, which was slightly below the EU average of 9.85 pmp (44, 45). Accordingly, the number of patients transplanted from living donors accounted only for 9.5% of all patients transplanted (44). However, this program has grown considerably over the past decade and is complemented by an active kidney exchange program that performed its first transplants in 2013 (17, 34, 45, 46).

Similarly, DCD only plays a limited role in the Portuguese transplant system. In 2013, the Ministry of Health implemented DCD in cooperation with the professional societies of intensive care medicine, nursing, and emergency services. However, due to ethical considerations brought forward by the National Council of Ethics in Life Sciences and a strong role of the National Institute for Medical Emergency, it was decided to restrict DCD to uncontrolled DCD (47). Hence, there have only been pioneering uDCD efforts in four University Hospitals that have the infrastructure to perform normothermic regional perfusion and a close connection to the national transplant infrastructure (48, personal correspondence). As a result, Portugal performed 17 uncontrolled donations after circulatory death, accounting for approximately 6% of all donations in 2021 (49). Currently, legislation for controlled donation after circulatory death is under review and the Ministry of Health has started a national consensus process that might permit controlled DCD in the future (personal correspondence).

Taken together, both living donation and DCD only play a minor role in the Portuguese transplant system. However, both areas have been identified as areas of reform and will potentially become more important in the near future.

##### Response to the COVID-19 Pandemic

The responses of the Portuguese health system to the COVID-19 pandemic included far-reaching containment measures involving two full lockdowns, a mobilization of resources to support the health system, a hierarchical vaccination program, and digital measures to improve contact tracing and telemedicine (3). While the 2021 vaccination rates in Portugal exceeded the European average and telemedicine consultations rose during the pandemic waves, digital contact tracing measures were not as successful largely as a result of the ongoing public debate on data protection (3). Overall, measures taken by the Portuguese health system reduced peak viral transmission, but were not sufficient to prevent over-stretching of intensive care capacity during the second wave of the pandemic (3).

As might be expected, transplantation rates fell sharply following both the first as well as second waves of the pandemic (in early 2020 and late 2020/early 2021, respectively) (50). This resulted in a 19% decrease in the number of transplantations performed per million population compared to 2019 (5, 44, 51). The rate of reduction was higher than that experienced by other European countries even after accounting for the general impact of the pandemic (as indicated by death rates secondary to COVID-19) (50). However, daily transplant activity in Portugal rapidly increased following each of the two pandemic peaks indicating capacity to recover quickly from external shocks (52). Additionally, the increase that followed the second wave began from a higher point and increased more rapidly than after the first wave. These results suggest that a “learning effect” had a significant impact on the rate at which transplant capacity could be re-built.

Collectively, these developments document the notable impact of the COVID-19 pandemic on transplantation rates in Portugal. This was accompanied by a rapid recovery of transplant capacity once peak virus transmission had passed.

## Discussion

Consistent with findings presented in previous analyses of the Portuguese organ donation and transplantation program, the results of our study demonstrate the importance of a set of core reforms that were successfully adapted from the Spanish model. In general, an integrated national health system that provides broad coverage, sufficient funding, technical infrastructure, comparatively low labor costs, the potential to provide financial incentives to participating clinical staff, and similar baseline demographics have been suggested to facilitate adoption of the Spanish model (53). These factors may have also influenced the successful adoption of the Spanish model in Portugal.

Most healthcare facilities in Portugal are either part of the national health service or regulated and connected with the Ministry of Health *via* negotiation (54). Although financial coverage for healthcare in Portugal is somewhat lower than that provided in other European countries, virtually the entire population is covered by the Portuguese National Health Service (Serviço Nacional de Saúde; SNS) or insurance schemes that offer an SNS-based broad benefits package (3). The organizational structure of the SNS includes a single central national authority and multiple regional authorities and thus mirrors the general structure of the health system in Spain (54, 55).

Portugal has a relatively large number of physicians who are paid comparatively moderate salaries in relation to the average incomes reported nationwide (3, 56). According to Matesanz et al. (53), this scenario will facilitate the implementation of financial incentives and thus the successful adaption of the Spanish model (53).

Portugal is home to comparatively few intensive care unit (ICU) beds given the size of its population. When donation rates in Portugal peaked in 2010, the number of available ICU beds *per capita* was at the low end of the spectrum in Europe; the number of critical care beds *per capita* was around half that reported to be available in Spain (4). However, the number of ICU beds has increased as part of the strategies to deal with the COVID-19 pandemic. As a result, latest available data shows a total number of 891 ICU-beds corresponding to approximately 8.6 ICU-beds per 100.000 inhabitants (57, 58). This number is still significantly below the Organization for Economic Co-operation and Development (OECD) average, but only slightly below that reported in Spain (59). Therefore, ICU capacity may have been a limitation for Portugal in the past, but the recent growth in capacity might facilitate organ donation and transplantation in the future.

Age and health status of the general population and the number of fatal accidents are relevant parameters in organ donation and transplantation (53). In Portugal, the fraction of the population over 65 years of age continues to grow and 50% of these individuals have been diagnosed with at least one chronic condition (3, 60). Accordingly, the number of organ donors that died due to medical reasons (most prominently cardiovascular events) has increased over time (61).

At the same time, there has been a significant reduction in the number of fatal traffic accidents in the country over the past 20 years (62). Accordingly, slightly fewer organ donors have died of trauma in the past 10 years, accounting for only 20% of all organ donors in 2021.

In summary, an aging demographic in Portugal may serve to maintain a sufficient pool of potential donors, compensating for a decrease in trauma-related deaths. However, donations from elderly, comorbid donors may lead to challenges regarding the quality of organs in the future.

Finally, largely due to their geographic proximity, Spain and Portugal share numerous cultural features, including a similar history of governmental and economic development in the 19th through the 21st centuries (63) as well as parallel views on end-of-life care that are linked to strong family and community ties and the influence of Catholicism (64). These similar cultural contexts have likely supported the successful adaptation of Spanish policies that are currently in use in the Portuguese program.

Taken together, Portugal fulfills many criteria that have been suggested to facilitate the adoption of the Spanish model. This may provide at least a partial explanation of Portugal’s success compared to that experienced by European countries of similar size and with similar financial resources.

The findings presented in this study are based on a comprehensive contemporary review of the Portuguese organ donation and transplantation program. Our results reflect a broad range of evidence, including a thorough search of both the academic and grey literature followed by expert review and synthesis using a multi-dimensional framework approach. The study summarizes key features of the program, including some findings that were described in previous analyses. Our study also highlights several complementary aspects and recent developments in Portugal. However, among the limitations of this study, we were unable to explore the post-transplant follow-up or research and development and focused on the time period prior to the disruption of COVID-19. Future studies of the Portuguese organ donor and transplant system might focus on post-transplant follow-up, and the role of professional societies in Portugal and might cover more recent developments in more detail.

In conclusion, Portugal has become a world leader in organ donation and transplantation largely because of its successful adaptation and adoption of the main components of the highly effective Spanish system. Thus, the Portuguese experience provides a valuable example to other countries with modest resources seeking to achieve higher rates of donation and transplantation. As outlined above, the Portuguese case provides evidence to support the implementation of a specific set of reforms that may also be used in other settings and contexts. However, Portugal has benefitted from several conditions that have facilitated its strong partnership with Spain and the successful implementation of the Spanish model. These advantages include its close cultural and geographical proximity in the Iberian peninsula as well as a similar economy and health system structure. We recognize that this situation is unique and that other countries may not necessarily have the capacity to build on these specific factors. Thus, while countries seeking to reform their national transplant systems should certainly be inspired by the Portuguese example, they also need to accommodate for local context and build on their individual strengths.

## Data Availability

The original contributions presented in the study are included in the article/supplementary material, further inquiries can be directed to the corresponding author.
